# Patterns and predictors of thromboembolic events among patients with gastric cancer

**DOI:** 10.1038/s41598-020-75719-w

**Published:** 2020-10-28

**Authors:** Hikmat Abdel-Razeq, Rawan Mustafa, Baha’ Sharaf, Abdallah Al-Tell, Dina Braik, Khaled Ashouri, Zaid Omari, Razan Mansour, Jamil Qarqash, Hanin Shaqboua, Saba Jaradat, Kholoud Al-Qasem, Rayan Bater

**Affiliations:** 1 Department of Internal Medicine, King Hussein Cancer Center, Queen Rania Al Abdullah Street, P.O. Box: 1269, Amman, 11941 Jordan; 2 School of Medicine, University of Jordan, Amman, Jordan; 3 Department of Radiology, King Hussein Cancer Center, Amman, Jordan; 4 Department of Medicine, Istishari Hospital, Amman, Jordan

**Keywords:** Cancer, Oncology

## Abstract

Patients with gastric cancer are at higher risk for venous thromboembolic events (VTE). Majority of such patients are treated in ambulatory settings where thromboprophylaxis is not routinely offered. In this study, we report on VTE rates and search for predictors that may help identify patients at higher risk to justify VTE-prophylaxis in ambulatory settings. Patients with pathologically-confirmed gastric adenocarcinoma were retrospectively reviewed for VTE detected by imaging studies. Clinical and pathological features known to increase the risk of VTE were studied. Khorana risk assessment model was applied on patients receiving chemotherapy. A total of 671 patients; median age 55 years, were recruited. VTE were diagnosed in 150 (22.4%) patients, including 42 (28.0%) pulmonary embolism and 18 (12.0%) upper extremity deep vein thrombosis (DVT). Majority (> 80%) developed VTE while in ambulatory settings and none had been on thromboprophylaxis. Rate was higher (27.1%) among 365 patients with metastatic compared to 16.7% among 306 patients with nonmetastatic disease, p = 0.001. Patients with metastatic disease who received multiple lines of chemotherapy (n = 85) had significantly higher rate of VTE compared to those who received a single line; 48.2% versus 19.4%, p < 0.001. Among the whole group, Khorana risk score, age, gender, smoking and obesity had no impact on VTE rates. Patients with metastatic gastric cancer, especially when treated with multiple lines of chemotherapy, are at a significantly higher risk of VTE. Khorana risk score had no impact on VTE rates. Thromboprophylaxis in ambulatory patients with metastatic gastric cancer worth studying.

## Introduction

Compared to other solid tumors, patients with gastric cancer are more prone to venous thromboembolic events^1,2^. Depending on the clinical characteristics of patients enrolled, the incidence varies significantly between studies. In one retrospective study of 112 patients; 59% with advanced-stage disease, VTE was encountered in 9.0%^3^. In a larger retrospective study that included over 2000 patients with gastric cancer, the 2-year cumulative incidences of all VTE events were as low as 0.5% in patients with stage-I disease to as high as 24.4% in those with stage IV (M1) disease^4^. However, other Asian studies had reported significantly lower rates; a larger South-Korean retrospective analysis of over 3000 patients with advanced gastric cancer reported a lower incidence of 3.5%^5^.

Thrombosis in cancer patients in general and gastric in particular, is not without major complications. Those who survival it, may run into major complications. Pulmonary embolism and subsequent pulmonary hypertension can be devastating^6,7^. Post-thrombotic syndrome with its poor mobility and intractable pain may negatively affect their quality of life^8^.

The process of full anticoagulation may be complicated by minor and major bleeding and may interrupt or delay surgical interventions or the administration of chemotherapy. Additionally, several studies had shown that the survival of patients with gastric cancer who develop VTE is significantly lower than those without^9,10^. In one recently published study from the RIETE registry that included 612 patients with gastric cancer and VTE, the overall mortality at 6 months was 44.4% and recurrent thromboembolic events were diagnosed in 6.5% of patients^11^. A multivariate analysis of the previously cited study, had shown that the development of extremity DVT or PE were significant predictors of early death when compared to those with no thrombosis^4^.

Thromboprophylaxis is strongly recommended and commonly practiced among most hospitalized patients with cancer and an acute medical illness^12^. However, majority of cancer patients, including those with gastric cancer, are treated and managed in ambulatory settings where VTE prophylaxis is not routinely practiced^13^. Several risk assessment models (RAM) had tried to link tumor- , patient- and treatment-related factors to the risk of VTE^14^. None of these models, including the Khorana^15^, and COMPASS^16^, were gastric cancer specific. Lack of strong evidence to routinely offer VTE prophylaxis for ambulatory cancer patients, and the fear of bleeding complications had resulted in higher rate of thromboembolic events.

In this study, we review our experience of VTE among patients with gastric cancer, mostly ambulatory, trying to identify factors that may increase the risk of VTE to levels high-enough to recommend VTE prophylaxis in ambulatory settings.

## Patients and methods

Medical records and hospital database of adult patients (≥ 18 years old) with pathologically-confirmed gastric adenocarcinoma; diagnosed, treated and followed at our institution between January 2007 and December 2017 were retrospectively reviewed. Only VTE confirmed by imaging studies were included. Clinical and pathological features known to increase the risk of VTE, including risk factors used to calculate the Khorana risk score were collected; such risk factors include the presence of central venous catheter (CVC), hemoglobin (Hb) level, platelet and white blood cell (WBC) counts, disease stage, and body mass index (BMI). Pharmacy database was also searched for any anticoagulant therapy for all patients enrolled.

Thrombosis was considered chemotherapy-related if diagnosed any time after the first dose and up to 4 weeks after the last. Ambulatory VTE was defined as those occurring in outpatient settings or diagnosed more than 4 weeks of the most recent hospitalization. Deep venous thrombosis was diagnosed by doppler ultrasound while PE was diagnosed by CT angiogram. Major bleeding, as a complication of anticoagulation, was defined as fatal bleeding and/or symptomatic bleeding in a critical organ and/or bleeding causing a fall in hemoglobin level of ≥ 2.0 g/dL, or leading to transfusion of two or more units of red blood cells. The study was approved by the King Hussein Cancer Center's institutional review board (IRB).

### Statistical analysis

The primary objectives were to determine the incidence and characteristics of VTE in adult patients diagnosed with gastric cancer. The secondary objective was to analyze the importance of the tumor, patients and treatment’-related characteristics in predicting the occurrence of thrombosis. Descriptive statistics were performed for patients’ clinical and pathological characteristics. Data was tabulated and described by ranges, medians and categorial variables were expressed as percentages (%). Variables and VTE incidences were examined for correlation.

Univariate analysis was done for patients’ data using chi-square test. Factors were categorized into risk groups and included in multivariate analysis using logistic regression analysis. A value of P < 0.05 was considered statistically significant. In result, the association between studied variables and occurrence of VTE was evaluated. Following this analysis, it was hypothesized that particular group(s) of patients with specific pathological or clinical features can be identified as high risk for VTE.

### Ethical approval

All procedures performed in the study involving human participants were in accordance with the ethical standards of the institutional research committee (IRB) at King Hussein Cancer Center and with the 1964 Helsinki declaration and its later amendments.

### Informed consent

Written informed consent was waived by the ethics committee at King Hussein Cancer Center.

### Conference presentation

Manuscript was presented at the annual meeting of the International Society on Thrombosis and Haemostasis (ISTH) (Virtual Congress), July 12–14, 2020.

## Results

During the study period, a total of 671 patients (59.6% males), median age (range) 55 (18–95) years fulfilled our inclusion criteria and were enrolled. More than half (n = 365, 54.4%) of the patients had metastatic disease at diagnosis and were treated with palliative intent. Among the 306 patients with early-stage disease, 291 (95.1%) underwent gastric surgery including 99 (34.0%) with total gastrectomy. Neoadjuvant chemotherapy was given to 84 (27.5%) while 182 (59.5%) patients were treated with adjuvant chemotherapy. A central venous access (CVC) was inserted to deliver chemotherapy for patients with early- and advanced-stage disease (n = 86, 12.8%), details of patients’ demographics and characteristics are presented in Table 1.

**Table 1:** Patients’ clinical characteristics (n = 671).

Characteristics	Number	Percentage
**Age (years)**		
**Median (range)**	55 (18–95)	
**Sex**		
Male	400	59.6
Females	271	40.4
**Body mass index (BMI) (kg/m**^**2**^**)**		
**Median (range)**	24 (8.2–55.2)	
< 25	344	51.3
25–29.9	176	26.2
≥ 30	93	13.9
**Smoking history**		
Current smoker	174	25.9
Former-smoker	124	18.5
Never smoked	323	48.1
Unknown	50	7.5
**Stage**		
Non-metastatic	306	45.6
Metastatic	365	54.4
**Surgery**		
All surgeries	291	43.4
Total gastrectomy	99	14.8
Partial gastrectomy	44	6.6
Distal subtotal	123	18.3
Other procedure	25	3.7
No surgery	380	56.6
**Central venous catheter (CVC)**	86	12.8
**Chemotherapy**		
All chemotherapy	570	84.9
Neoadjuvant	84	12.5
Adjuvant	182	27.1
Palliative	304	45.3

During the first 12 months of follow up, 150 (22.4%) patients had a confirmed diagnosis of VTE, including 42 (28.0%) PE and 18 (12.0%) upper extremity DVT. All patients were treated with enoxaparin, a low molecular weight heparins (LMWH), at the usual dose of 1.0 mg/kg subcutaneously twice daily. None of the patients received warfarin or direct oral anticoagulants (DOAC). Anticoagulation was complicated by bleeding in 14 (9.3%) patients; two were major but not fatal, while 6 (4.0%) had thrombocytopenia and 33 (22.0%) had recurrent thrombosis.

Majority of the patients (n = 132, 88.0%) had their VTE while having active disease; 94 (62.7%) within 4 weeks from chemotherapy. Only 25 (16.7%) patients had their clots while in-patient or within 4 weeks of recent hospitalization, while majority (83.3%) developed VTE while in ambulatory settings and none were on thromboprophylaxis, Table 2. Only 7 (4.7%) of the 150 VTE were related to surgery and occurred within 30 days of surgical intervention.

**Table 2:** Venous thromboembolism (n = 150).

Clinical variables	Number of patients	Percentage
**Disease status at time of VTE**		
Disease-free	18	12.0
Active disease	132	88.0
Chemotherapy within 30 days	94	62.7
VTE within 30 days of hospitalization	25	16.7
**VTE site**^**a**^		
Lower extremity	35	23.3
Upper extremity	18	12.0
PE	42	28.0
Others	57	38.0

^a^Total > 150 as some patients had multiple sites; VTE: Venous Thromboembolism.

Venous thromboembolism was diagnosed in 99 (27.1%) patients with metastatic disease compared to 51 (16.7%) patients with early-stage disease, p = 0.001. Chemotherapy was given to 255 (69.9%) patients with metastatic disease; 74 (29.0%) had VTE compared to 25 (22.7%) of 110 patients who did not receive chemotherapy, p = 0.215. Rate of VTE was significantly higher (n = 41, 48.2%) among a group of 85 patients who had multiple lines (regimens) of chemotherapy, compared to 33 (19.4%) of 170 patients who had a single-line of chemotherapy, p < 0.001. No difference in VTE rates were noted between patients who received cisplatin-based regimen DCF (docetaxel, cisplatin, 5-FU) and those who received other chemotherapy regimens, Table 3. On the other hand, VTE rate was significantly lower among a group of 101 patients with early-stage disease who never received chemotherapy compared to 203 patients who did; 9.9% versus 20.2% (p = 0.022), respectively.

**Table 3:** Venous thromboembolism rates among patients with metastatic disease, n = 365.

	Number of patients	VTE cases (number, %)	p-value
**Chemotherapy**			
No	110	25 (22.7%)	0.215
Yes	255	74 (29.0%)	
**Lines of chemotherapy**			
Single-line	170	33 (19.4%)	< 0.001
Multiple-lines	85	41 (48.2%)	
**Chemotherapy regimen**			
DCF	166	43 (25.9%)	0.681
Others	199	56 (28.1%)	

DCF: Docetaxel, Cisplatin, 5-FU Regimen; VTE: Venous Thromboembolism.

We also applied the Khorana risk assessment model on patients about to receive chemotherapy; VTE rate was 30.2% among a group of 202 with high-risk scores compared to 26.5% among 234 patients with intermediate-risk score, p = 0.194. Additionally, rates of VTE were similar in active smokers (20.1%) and those who never smoked (21.7%), p = 0.485 and in obese (BMI ≥ 30) patients (29.0%) compared to 21.3% among those with BMI < 30, p = 0.280, Table 4.

**Table 4:** Rates of venous thromboembolism by subgroup.

Risk factors	Groups	Total	VTE (n)	VTE (%)	p-value
Age at diagnosis (years)	< 50	254	51	20.1	0.119
	≥ 50	417	99	23.7	
Sex	Male	400	83	20.8	0.093
	Female	271	67	24.7	
Body mass index (BMI)	< 30	520	123	23.7	0.280
	≥ 30	93	27	29.0	
Smoking	Current smoker	174	35	20.1	0.485
	Never smoked	323	70	21.7	
	Former smoker	124	29	23.3	
Disease stage	M0	306	51	16.7	0.001
	M1	365	99	27.1	
Khorana risk score^a^	Intermediate	234	62	26.5	0.194
	High	202	61	30.2	

^a^From a total of 436 patients prior to chemotherapy; VTE: Venous Thromboembolism.

We also applied a univariate analysis to further address the risk of VTE. The effect of age, gender, BMI, smoking history, disease stage, the need for multiple lines of chemotherapy and surgery were studied. Only disease stage and the need for multiple lines of chemotherapy were significantly associated with VTE. Both factors remained significant in multivariate analysis with Odd Ratio (OR) of 2.24 [95% Confidence Interval (CI) 1.423, 3.528, P < 0.001] and 2.51 [95% CI 1.428, 4.420, P = 0.001], respectively. BMI and smoking history were not significant, Table 5.

**Table 5:** Analysis of independent risk factors (multivariate).

Variables	Odd ratio (OR)	95% confidence interval (CI)	P-value
Body mass index (BMI)	1.45	0.887, 2.438	0.168
Smoking history	0.96	0.647, 1.440	0.861
Disease metastasis	2.24	1.223, 3.528	0.008
Multiple-lines chemotherapy	2.51	1.428, 4.420	0.001

## Discussion

Venous thromboprophylaxis in patients with gastric cancer is always a challenge. Balancing the known high-risk of thrombosis with the high-risk of bleeding associated with anticoagulation is not easy. Patients with gastric cancer are at higher risk of bleeding compared to other patients with other solid tumors. Efforts pushing for more prophylaxis are always pulled back when faced with higher rates of bleeding. However, the risk of bleeding following full-dose anticoagulation is known to be significantly higher than the risk associated with prophylactic dose. Such fact might not be in the mind of most practicing physicians when making such decisions. Therefore, we need to factor-in and may accept the few episodes of bleeding, mostly minor, encountered in most of VTE prophylaxis trials. Such bleeding, in quantity and frequency, is significantly lower than those associated with full-dose therapeutic anticoagulation, if we wait for the thrombus to happen. Risk assessment models, in high risk tumors like gastric cancer, should calculate the gain of preventing VTE against the net loss due to bleeding in both occasions; therapeutic versus prophylactic dose of anticoagulation.

Our VTE rate of 22% is higher than several previously published studies and can be explained, at least in-part, by the higher percentage of patients with metastatic disease in our cohort (54%), but other contributing factors cannot be ruled out. Several other findings in our study worth highlighting and may help us make decisions on VTE prophylaxis for ambulatory patients:

First, 87% of our confirmed VTE episodes were among patients with active disease at time of diagnosis. This is expected given the hypercoagulable state that is known to be induced by adenocarcinomas in general, and gastric cancer in particular^17^. Additionally, patients with active disease are likely to be treated with chemotherapy which is by itself can increase the risk of VTE. Additional factors that may increase such risk include poor performance status and frequent hospitalizations associated with active disease state.

Second, the fact that those with early stage disease who never had any form of chemotherapy had a relatively low rate of VTE (9.9%) might indicate that such patients, without the need to apply any risk assessment model, be considered not at high-enough risk to offer VTE prophylaxis while ambulatory.

Third, patients who had multiple lines of chemotherapy had significantly higher VTE rate than those who only had one. This could be a surrogate marker of disease activity and might also reflect poor performance status and ambulatory level.

Fourth, chemotherapeutic agents used in gastric cancer are also thought to affect the risk of VTE. A prospective controlled trial of 964 patients treated with epirubicin, platinum and fluoropyrimidine combination chemotherapy in stage 3 and 4 gastric cancer found more thromboembolic events in the cisplatin group compared with the oxaliplatin groups (15.1% versus 7.6%; p = 0.0003)^18^. We have previously reported our experience with VTE associated with cisplatin-based chemotherapy in a group of 1677 patients with various solid tumors including 191 patients with gastric cancer. Among the whole group, thromboembolic events were confirmed in 6.6% but was highest (20.9%) in patients with gastric cancer. In multivariate analysis, significantly higher rates of thrombosis were associated with gastric, as the primary tumor, and advanced-stage disease^19^. More recently, the FLOT4 (fluorouracil plus leucovorin, oxaliplatin and docetaxel) trial also showed lower risk of thromboembolic events in the cisplatin-free regimen (FLOT4) compared to other cisplatin-containing regimens^20^. An increase in the incidence of VTE was also observed in patients with gastric cancer receiving other agents like irinotecan or bevacizumab in locally advanced and metastatic settings, reaching up to 25% of patients in the study^21^. The high VTE rate among our cohort may explain the lack of difference between cisplatin-based regimen and others in our study.

Last, despite having gastric cancer among the highest risk tumors in Khorana RAM, the model couldn’t separate patients in our study into different risk levels. The high VTE rates among our cohort may explain such finding. Additionally, as defined by Khorana RAM and just because of type of their cancer, patients with gastric cancer are either at intermediate or high risk but not low risk (Table 6).

**Table 6:** Khorana risk assessment model.

Features	Risk score
1. Site of cancer	
Very high risk (stomach, pancreas)	2
High risk (lung, lymphoma, gynecologic, bladder, testicular)	1
2. Hb < 10 gm/dL or use of red cell growth factors	1
3. Prechemotherapy leukocyte count > 11 × 10^9^/L	1
4. Prechemotherapy platelet count ≥ 350 × 10^9^/L	1
5. BMI ≥ 35 kg/m^2^	1

BMI: body mass index.

Risk groups: Low risk = 0, Intermediate risk = 1–2, High risk ≥ 3.

Our study is not without limitations; we have not addressed underlying illnesses and comorbidities which may increase the rate of VTE. Though our data is derived from a single institution, the large number of patients included minimize selection bias. Our findings should highlight the need for a special RAM for patients with gastric cancer. Such model should take into consideration the stage of the disease, number of lines of chemotherapy and specific chemotherapeutic agents used.

## Conclusions

Patients with gastric cancer have notably high rate of VTE especially those with metastatic disease when treated with multiple lines of chemotherapy. Khorana risk score, age, gender, smoking and obesity had no impact on VTE rates. Thromboprophylaxis in ambulatory patients with gastric cancer worth more studying.

## References

[1] Sørensen HT, Mellemkjær L, Olsen JH, Baron JA (2000). Prognosis of cancers associated with venous thromboembolism. N. Engl. J. Med..

[2] Tanizawa Y, Bando E, Kawamura T, Tokunaga M, Makuushi R (2016). Prevalence of deep venous thrombosis detected by ultrasonography before surgery in patients with gastric cancer: A retrospective study of 1140 consecutive patients. Gastric Cancer.

[3] Fuentes HE, Oramas DM, Paz LH (2017). Venous thromboembolism is an independent predictor of mortality among patients with gastric cancer. J. Gastrointest. Cancer..

[4] Lee K, Bang S, Kim S, Lee H, Shin D, Koh Y (2009). The incidence, risk factors and prognostic implications of venous thromboembolism in patients with gastric cancer. J. Thromb. Haemost..

[5] Kang M, Ryoo B, Ryu M, Koo D, Chang H, Lee J (2012). Venous thromboembolism (VTE) in patients with advanced gastric cancer: An Asian experience. Eur. J. Cancer.

[6] Klok FA, Couturaud F, Delcroix M, Humbert M (2020). Diagnosis of chronic thromboembolic pulmonary hypertension after acute pulmonary embolism. Eur. Respir. J..

[7] McCabe C, Dimopoulos K, Pitcher A, Orchard E, Price L, Kempny A (2020). Chronic thromboembolic disease following pulmonary embolism: Time for a fresh look at old clot. Eur. Respir. J..

[8] Rabinovich A, Kahn SR (2018). How I treat the post-thrombotic syndrome. Blood.

[9] Horsted F, West J, Grainge M (2012). Risk of venous thromboembolism in patients with cancer: A systematic review and meta-analysis. PLoS Med..

[10] Donnellan E, Khorana AA (2017). Cancer and venous thromboembolic disease: A review. Oncologist.

[11] Majmudar K, Golemi I, Tafur A, Toro J, Visonà A, Falgá C (2020). Outcomes after venous thromboembolism in patients with gastric cancer: Analysis of the RIETE Registry. Vasc. Med..

[12] Key N, Khorana A, Kuderer N, Bohlke K, Lee A, Arcelus J (2020). Venous thromboembolism prophylaxis and treatment in patients with cancer: ASCO clinical practice guideline update. J. Clin. Oncol..

[13] Abdel-Razeq H, Mansour A (2017). Venous thromboembolism prophylaxis for ambulatory cancer patients, can we do better?. J. Thromb. Thrombolysis.

[14] Fuentes H, Paz L, Wang Y, Oramas D, Simons C, Tafur A (2017). Performance of current thromboembolism risk assessment tools in patients with gastric cancer and validity after first treatment. Clin. Appl. Thromb. Hemost..

[15] Khorana A, Kuderer N, Culakova E, Lyman G, Francis C (2008). Development and validation of a predictive model for chemotherapy-associated thrombosis. Blood.

[16] Gerotziafas G, Taher A, Abdel-Razeq H, AboElnazar E, Spyropoulos A, El Shemmari S (2017). A predictive score for thrombosis associated with breast, colorectal, lung, or ovarian cancer: The prospective COMPASS–cancer-associated thrombosis study. Oncologist.

[17] Hisada Y, Mackman N (2017). Cancer-associated pathways and biomarkers of venous thrombosis. Blood.

[18] Starling N, Rao S, Cunningham D, Ivsen T, Nicolson M, Coxon F (2009). Thromboembolism in patients with advanced gastroesophageal cancer treated with anthracycline, platinum, and fluoropyrimidine combination chemotherapy: A report from the UK National Cancer Research Institute Upper Gastrointestinal Clinical Studies Group. J. Clin. Oncol..

[19] Abdel-Razeq H, Mansour A, Abdulelah H, Al-Shwayat A, Makoseh M, Ibrahim M (2018). Thromboembolic events in cancer patients on active treatment with cisplatin-based chemotherapy: Another look!. Thromb. J..

[20] Al-Batran SE, Homann N, Pauligk C, Goetze TO, Meiler J, Kasper S (2019). Perioperative chemotherapy with fluorouracil plus leucovorin, oxaliplatin, and docetaxel versus fluorouracil or capecitabine plus cisplatin and epirubicin for locally advanced, resectable gastric or gastro-oesophageal junction adenocarcinoma (FLOT4): A randomised, phase 2/3 trial. Lancet.

[21] Shah M, Ilson D, Kelsen D (2005). Thromboembolic events in gastric cancer: High incidence in patients receiving irinotecan- and bevacizumab-based therapy. J. Clin. Oncol..

